# Evaluation of ChatGPT Performance on Emergency Medicine Board Examination Questions: Observational Study

**DOI:** 10.2196/67696

**Published:** 2025-03-12

**Authors:** Mila Pastrak, Sten Kajitani, Anthony James Goodings, Austin Drewek, Andrew LaFree, Adrian Murphy

**Affiliations:** 1 School of Medicine, University College Cork, Cork, Ireland; 2 Department of Emergency Medicine, Johns Hopkins University, Baltimore, MD, United States; 3 Department of Emergency Medicine, University of California, San Diego, 200 W. Arbor Dr. #8676, San Diego, CA, 92103, United States, 1 6198319501; 4 Department of Emergency Medicine, Cork University Hospital, Cork, Ireland

**Keywords:** artificial intelligence, ChatGPT-4, medical education, emergency medicine, examination, examination preparation

## Abstract

**Background:**

The ever-evolving field of medicine has highlighted the potential for ChatGPT as an assistive platform. However, its use in medical board examination preparation and completion remains unclear.

**Objective:**

This study aimed to evaluate the performance of a custom-modified version of ChatGPT-4, tailored with emergency medicine board examination preparatory materials (Anki flashcard deck), compared to its default version and previous iteration (3.5). The goal was to assess the accuracy of ChatGPT-4 answering board-style questions and its suitability as a tool to aid students and trainees in standardized examination preparation.

**Methods:**

A comparative analysis was conducted using a random selection of 598 questions from the Rosh In-Training Examination Question Bank. The subjects of the study included three versions of ChatGPT: the Default, a Custom, and ChatGPT-3.5. The accuracy, response length, medical discipline subgroups, and underlying causes of error were analyzed.

**Results:**

The Custom version did not demonstrate a significant improvement in accuracy over the Default version (*P*=.61), although both significantly outperformed ChatGPT-3.5 (*P*<.001). The Default version produced significantly longer responses than the Custom version, with the mean (SD) values being 1371 (444) and 929 (408), respectively (*P*<.001). Subgroup analysis revealed no significant difference in the performance across different medical subdisciplines between the versions (*P*>.05 in all cases). Both the versions of ChatGPT-4 had similar underlying error types (*P*>.05 in all cases) and had a 99% predicted probability of passing while ChatGPT-3.5 had an 85% probability.

**Conclusions:**

The findings suggest that while newer versions of ChatGPT exhibit improved performance in emergency medicine board examination preparation, specific enhancement with a comprehensive Anki flashcard deck on the topic does not significantly impact accuracy. The study highlights the potential of ChatGPT-4 as a tool for medical education, capable of providing accurate support across a wide range of topics in emergency medicine in its default form.

## Introduction

### Background

The integration of artificial intelligence (AI) into medical education represents a frontier with the potential to significantly enhance learning outcomes and examination preparation strategies [1-5]. This advancement comes at a crucial time when the medical field faces the dual challenges of rapidly evolving knowledge bases and the increasing complexity of patient care. Among the AI tools making strides in educational contexts, ChatGPT has emerged as a notable platform [6]. Its ability to generate human-like text based on a vast database of information has sparked interest in its application for medical board examination preparation.

Previous studies have shown mixed results regarding the effectiveness of AI in medical education, with certain limitations identified in AI’s ability to replicate the depth of understanding needed to answer questions correctly in high-stakes examinations [7-12]. Building upon this background, our study seeks to determine whether a targeted enhancement of ChatGPT-4 can increase the accuracy of the model in answering board examination questions, particularly for the American Board of Emergency Medicine (ABEM) Examinations.

ChatGPT provides relatively accurate responses to questions in examinations such as the USMLE (United States Medical Licensing Examination) [13,14] and the ABFM (American Board of Family Medicine) examination [5]. This may instill the confidence in takers of these examinations to use ChatGPT as an additional tool to aid in preparation. For instance, when reviewing a question set, the trainee may use ChatGPT to provide the rationale for a correct answer or help them understand the questions that they responded incorrectly to. This provides the potential to streamline the preparation process by reducing the need to consult textbooks or internet-based resources, as retaining interaction with multiple sources, such as a validated question bank, flashcards, and ChatGPT, is likely to bolster confidence in the overall educational outcome [15]. Additionally, the functionality of ChatGPT enables the user to ask follow-up questions or for further clarification if the initial response is insufficient.

In the pursuit of enhancing the capabilities of ChatGPT-4 for emergency medicine board examination preparation, a comprehensive Anki deck was utilized as a resource for custom modification [16,17]. The specific Anki deck chosen, “The Emergency Medicine Residents’ Deck,” also called “Rob’s Emergency Medicine Deck” [18], is a collection of emergency medicine knowledge, aggregating content from various premade decks and covering a wide array of topics pertinent to the field.

The information within this deck is sourced from a variety of educational resources and study aids [18]. The deck’s development and maintenance are overseen by medical professionals, with a commitment to regular updates and improvements based on the latest research, peer-reviewed consensus, and user feedback.

### Rationale

Medical learners seem to generally have a positive view on generative AI [19-21]. Incorporating its potential with another popular and effective resource [22,23], Anki, could be useful to this population. The hypothesis driving this study posits that a ChatGPT-4 model, when enhanced with the comprehensive knowledge contained in this Anki deck, would outperform its standard counterpart in emergency medicine board examination preparation. This assumption is grounded in the belief that the deck’s content could significantly bolster the AI’s understanding and response accuracy to examination-relevant questions. Moreover, a positive outcome from this hypothesis could suggest that medical students who use this Anki deck for preparation could potentially be equipped with all the knowledge to excel in the board examination.

The Anki deck was chosen as it is designed to be a comprehensive resource. Additionally, Anki has become one of the most popular study methods among trainees and medical students. The approach of spaced repetition is particularly useful in helping people recall information. While an AI model would not engage in spaced repetition, the content of the decks can be used to train the AI. By using this method, it can allow us to evaluate the performance of ChatGPT when provided with a widely used, evidence-based resource. Relative to other resources such as textbooks, an Anki user endeavors to recall every piece of information in the deck, while a textbook is generally not used in the same way.

### Aims and Objectives

This study aimed to explore the efficacy of ChatGPT-4, specifically a custom-modified version tailored with specialized preparatory materials, in the context of emergency medicine board examination preparation. The objectives of this work were to: (1) evaluate the accuracy of ChatGPT-3.5 (released in 2022) in answering board examination style questions, (2) assess the baseline capabilities of the standard ChatGPT-4 model (released in 2023) in answering board examination questions accurately and consistently, and (3) evaluate whether a version custom-trained with a comprehensive flashcard resource exhibits superior performance. This comparison aimed to shed light on the potential of AI as a tool for medical education and identify pathways for its optimization in this domain.

## Methods

### Resources and Procedure

We used the Rosh In-Training Examination Question Bank, comprising 2000 questions, as the primary resource for questions. In order to customize ChatGPT-4 and transform it into a more specialized emergency medicine language model, “Rob’s Emergency Medicine Deck,” a comprehensive Anki deck for the ABEM Examinations, was converted to a TXT file and used to train the modified ChatGPT-4 model named “Emergency Medicine Residency Board Examination Expert.”

Questions were selected from the question bank by selecting the “unused questions” option during the creation of individual practice examination question sets to ensure random selection and no overlapping questions.

### Statistical Analysis

#### Sample Size

To examine if the sample size of 598 questions that were evaluated out of 2000 questions from the Rosh Review database is sufficient to make a conclusion about the performance of the two language models being equal, the following statistical assessment of the proportion of correct answers in each database was performed: the two-proportion *z* test was implemented to determine if there is a significant difference in error rates between the two language models; the alpha level of 0.05 was set to test the null hypothesis. The power was set at 0.80. The CIs for the difference between the two proportions were calculated; for the 5% significance level, a CI of 95% that included 0 would imply no significant difference between the error rates of the two language models.

The analysis showed that the two-proportion *z* score of approximately –0.073 corresponded to a *P* value of 0.942. Therefore, no statistically significant difference between the error rates indicates equal performance of the two language models. The *z* score close to 0 is also within the range of typical sampling variation. In addition, the CIs for the proportions of correct answers using the Wilson Score Interval were approximately 77.3% to 83.6% for Custom ChatGPT-4 versus 77.1% to 83.5% for Default ChatGPT-4. The CI for the differences between the two proportions ranged between –4.7% and 4.3%. This narrow difference between the two proportions included 0, further showing no significant difference in the performance of the two language models.

Hence, a sample size of 598 questions that represent 29.9% of the Rosh Review database is sufficient to reliably assess the performance of the two language models.

#### Comparative Analysis

The performance of both the default and enhanced ChatGPT-4 models was compared based on the number of correct and incorrect answers. The incorrect responses were categorized according to the reason for error (logical error, informational error, or other), an approach used in previous studies [5,24], and analyzed for patterns.

A logical error is when the response successfully identified the relevant information but failed to effectively transform it into an answer. For example, the model identifies that a patient is struggling with the consistent use of topical acne medications due to a busy schedule and yet selects the answer that is a daily treatment over a less frequent regimen.

An informational error is when ChatGPT missed a crucial detail, either contained within the question or from external sources that should be part of its expected knowledge base. For example, a young woman is seeking birth control with a history of deep vein thrombosis, yet it recommends the oral contraceptive pill when deep vein thrombosis is a contraindication.

All remaining errors that are not related to the nonadequate connection to information, had insufficient consideration of all elements of the information, or had an arithmetic mistake were classified as "other". For example, the model identifies that a patient has cardiac failure yet inaccurately classifies the patient per the New York Heart Association Classification.

#### Incorrect Response Analysis and Question Type Assessment

For each incorrect response, the explanation provided by ChatGPT-4 was quantified (as response length in characters without spaces). Incorrect questions were classified by type (cardiac emergencies, neurological emergencies, respiratory emergencies, etc) to identify specific areas of weakness.

#### Statistical Analysis and Data Manipulation

A combination of statistical tests and data manipulation techniques were employed, facilitated by Python. The data were managed and manipulated using Pandas [25], a Python library offering data structures and tools designed for efficient data manipulation and analysis. Tasks such as filtering data, computing descriptive statistics, and organizing data into contingency tables for further statistical testing were conducted.

For statistical analyses, several methods were employed to assess differences in performance between versions of ChatGPT. The McNemar test was carried out using the SciPy library [26] to compare paired nominal data across different subgroups. Additionally, for comparisons involving proportions, the proportions_*z* test function from the Statsmodels library [27], which provides comprehensive classes and functions for estimating different statistical models and performing statistical tests, was used.

Furthermore, the Wilcoxon signed-rank test, through the SciPy library, was applied for the analysis of paired proportions with nonparametric methods to assess the statistical significance of differences between the versions without assuming the normal distribution of the data. CIs for proportions were estimated using a normal approximation method, underlining the assumptions made regarding the distribution of the sample proportions.

### Ethical Considerations

As an observational study involving an AI system, there were no human or animal subjects, thus minimizing ethical concerns. Ethical approval was not required for this study in accordance with the criteria of the Clinical Research Ethics Committee of the Cork Teaching Hospitals, University College Cork.

## Results

### Data Collection

All results were collected from February 24, 2024 to March 13, 2024. The default ChatGPT-4 model was tested by manually entering a randomized selection of 598 questions from the Rosh In-Training Examination Question Bank. The ChatGPT-3.5 model was tested using a randomized selection of 269 questions from the same set of questions presented to the default ChatGPT-4 model.

### Comparison of Models

#### Percent of Questions Correct

Table 1 shows the performance of Custom ChatGPT-4, Default ChatGPT-4, and Default ChatGPT-3.5 on the randomized 598 question Rosh Review bank. Custom ChatGPT-4 and Default ChatGPT-4 answered 481 questions (80.4%, 95% CI 77.3% to 83.6%) and 480 questions (80.3%, 95% CI 77.1% to 83.5%) correct, respectively, with *P*=.61. These results indicate that the overall performance for correctly answering is similar between the two versions, with overlapping CIs, suggesting no significant difference in their ability. However, Custom ChatGPT-4 significantly outperformed ChatGPT-3.5 by 17.6% while Default ChatGPT-4 significantly outperformed Default ChatGPT-3.5 by 17.5% (*P*<.001 and *P*<.001, respectively).

**Table 1.** The performance of three language models on the American Board of Emergency Medicine examination using the Rosh Review question bank.

	Custom ChatGPT-4 (n=598)	Default ChatGPT-4 (n=598)	Default ChatGPT-3.5 (n=269)
Number of Correct Questions	481	480	169
Correct (%)	80.4	80.3	62.8

#### Length of Responses

The Custom ChatGPT-4 had significantly shorter response lengths, 929 (SD=408) characters without spaces versus 1371 (SD=444) characters without spaces for the Default ChatGPT-4 (*P*<.001). This suggests that Default ChatGPT-4 provided either more comprehensive or verbose responses.

### Responses by Discipline

In Table 2, we conducted a subgroup analysis to explore the performance of the Custom ChatGPT-4 and Default ChatGPT-4 versions across 15 different disciplines within emergency medicine. There were no statistically significant differences in the number of correct questions per discipline between Custom ChatGPT-4 and Default ChatGPT-4 in the 15 groups: 12/15 of the subgroups had *P*=1.0, except ear, nose, and throat (*P*=.23); obstetrics and gynecology (*P*=.50); and other (*P*=.77).

**Table 2.** Comparison of custom ChatGPT-4 and default ChatGPT-4 correct performance in Rosh Review subgroup analysis.

Subgroup	Custom ChatGPT-4 (n, %)	Default ChatGPT-4 (n, %)
Cardiology	81 (72.8)	81 (71.6)
Respirology	48 (70.8)	48 (73.5)
Neurology	33 (87.9)	33 (84.9)
Infectious Diseases	72 (84.7)	72 (83.1)
Gastrointestinal	51 (80.4)	51 (82.4)
Renal	15 (80.0)	15 (86.7)
Reproductive	9 (88.9)	9 (88.9)
Endocrine	23 (78.3)	23 (78.3)
Musculoskeletal	37 (73.0)	37 (73.0)
Ear, Nose, and Throat	26 (80.8)	26 (92.3)
Dermatology	16 (81.3)	16 (81.3)
Ophthalmology	20 (90.0)	20 (85.0)
Obstetrics and Gynecology	24 (87.5)	24 (79.2)
Oncology and Hematology	30 (86.2)	30 (90.0)
Other (Environmental)	113 (82.5)	113 (80.5)

### Error Type Analysis

In Table 3, the type of error made by the Custom ChatGPT-4 and Default ChatGPT-4 was evaluated. There was no significant difference between Custom ChatGPT-4 and Default ChatGPT-4 for logical error (75.2% vs 80.5%), informational error (12.0% vs 13.6%), or other (12.8% vs 5.9%), with *P*=.41, *P*=.87, and *P*=.11, respectively.

**Table 3.** Assessment of the type of error conducted in two language models.

Error type	Custom ChatGPT-4 (n, %)	Default ChatGPT-4 (n, %)
Logical error	88 (75.2)	95 (80.5)
Informational error	14 (12.0)	16 (13.6)
Other	15 (12.8)	7 (5.9)
Total	117 (100)	118 (100)

### Probability of Achieving a Passing Score

The passing probability of each ChatGPT model as predicted by the Rosh Review according to the individual ChatGPT performance was compared to the true performance of emergency medicine residents who wrote the ABEM in 2023. The newest ChatGPT models, ChatGPT-4 had a 99% chance of passing in both the Custom and Default versions. These were higher than the 85% probability of the default ChatGPT-3.5 version to pass and the 88% overall pass rate for the human counterparts. Notably, the human counterparts outperformed the ChatGPT-3.5 model.

## Discussion

### Principal Findings

A prominent characteristic highlighted through the development of ChatGPT is its capacity to grasp the context and key details that are pertinent to the discussed subject. Our study demonstrates that this capability is also applicable within the medical field by evaluating three versions of ChatGPT with the same data set. We found that both the custom and default models are highly likely capable of passing the ABEM written examination. This is supported by the Rosh Review [28], which had a predictive measure of passing the examination with the probability of passing at 98.8% accuracy; the Rosh Review found that both models had a 99% probability of passing. However, ChatGPT-3.5 had an 85% probability of passing. This prediction suggests that the enhancements made for the custom-modified version did not significantly improve accuracy over the default version of ChatGPT-4 and also shows that advancements made between ChatGPT versions have potential applications in the medical field. These findings imply that the core capabilities of ChatGPT-4 are already sufficiently advanced for tasks such as aiding in emergency medicine board examination preparation. Furthermore, the recorded national average pass rate for first-time test takers is 91%, with the 2023 pass rate being 88% [29], suggesting that ChatGPT-4 has an improved performance while ChatGPT-3.5 is less equipped compared to humans.

In addition, our results illustrate that both models had consistent performance across various medical disciplines and highlight the versatility of ChatGPT as an educational tool. This versatility is particularly relevant in the context of emergency medicine, where a broad spectrum of knowledge is required, and suggests that AI can offer comprehensive support across diverse subject areas. Additionally, the integration of an Anki deck into a ChatGPT-4 model could help identify the specific flashcards and topics that the learners should focus on, an area for future research.

### Comparison of Error Types and Response Length

The custom and default models had a similar level of drawing incorrect conclusions and omitting important components of questions, both of which hint at areas for improvement in both models. The high percentage of logical errors, compared to the other two errors, indicates that language models may not be particularly well suited in deductive reasoning [30]. It may be possible to address this by careful prompt engineering [31], for instance, instructing the model to follow a hierarchy of information sources to deliver the most reliable answers consistently. This is an area that could be the subject of further research.

Additionally, the response length analysis revealed that longer responses do not necessarily correlate with increased accuracy. Prompt engineering could be used to enhance the ease of learning by outlining a preferred explanation format. This finding has practical implications for the design of AI-driven study tools, suggesting that brevity, combined with accuracy, could enhance the efficiency of study sessions and information retention for learners. In contrast, it could be argued that longer responses reflect more comprehensive explanations. Future studies and particularly a qualitative analysis could be done to interrogate these hypotheses.

### Effect of Custom Training on Performance

The results underscore the rapid advancements in AI technology, particularly in natural language processing and knowledge retrieval, which have significant implications for medical education. The observed improvements from version 3.5 to the more recent iterations of ChatGPT reflect a trajectory in AI development that could increasingly support complex learning needs. This evolution underscores the potential of AI to become an increasingly valuable asset in educational settings [6,19], offering up-to-date knowledge and adaptive learning paths on balance with a general cautious optimism among medical professionals [32]. Despite the lack of observed benefit from custom modifications in this context, the findings highlight the critical role of up-to-date AI models in enhancing learning outcomes. Furthermore, the results illustrate that the untrained ChatGPT-4 has a higher likelihood of passing compared to human test takers, who extensively prepared for the board examinations, suggesting that, even without custom modifications, ChatGPT-4 has sufficient accuracy to serve as a customizable tutor.

Overall, while the investigation revealed no significant difference in performance accuracy between the custom-modified and default versions of ChatGPT-4, both showed considerable improvement over the older 3.5 version. These findings prompt a re-evaluation of the presumed advantage of tailoring AI through specific educational content, suggesting that the core capabilities of advanced AI models might already be sufficiently robust for some less highly subspecialized educational applications. Additionally, these findings promote investigation into future upcoming ChatGPT models to evaluate if their advancements have accelerated benefit in the medical field.

When evaluating the reason for the Custom model not being significantly better than the Default model, we must consider that the Default version has already been trained on sufficiently similar data that the information provided did not contribute anything new to the knowledge base. The need for AI to be trained on up-to-date data is well established [33]. A previous study has hypothesized that training the model on static knowledge could potentially be a limiting factor [5], the reason for this being that online resources can be constantly updated with the latest guidelines and treatments. Basing training on a well-maintained dynamic knowledge source such as UpToDate® (Wolters Kluwer) could potentially provide more useful outcomes. It seems that general medicine knowledge has been well incorporated into the training material for the ChatGPT-4 model, and this can explain the similar performance between the two versions of ChatGPT-4 we tested. However, for more niche and subspecialized fields, there may exist a more pronounced benefit, and this is something future works could explore.

This study reaffirmed the potential of AI, particularly ChatGPT-4, as a powerful tool in medical education [6,34], capable of supporting learners in high-stakes examination preparation without the need for specialized enhancements. It highlighted the importance of leveraging the inherent capabilities of advanced AI models and provided a foundation for further research into effective integration strategies in educational settings. As AI continues to evolve, its role in education is likely to expand, offering opportunities to enrich learning experiences and access to knowledge.

### Limitations

While this study provides valuable insights, it is not without limitations. The scope was restricted to emergency medicine, limiting the generalizability of the findings to other fields of medicine or education. Future research could explore the application of AI in different specialties to assess its versatility and effectiveness further.

Additionally, the study’s design focused on the efficacy of AI in answering board examination questions, which may not fully capture the nuances of applying that knowledge in clinical practice [35]. Further studies could investigate the impact of AI-assisted learning on clinical skills and decision-making processes [36,37]. The results of this study are not generalizable to the use of AI in contexts of medical education beyond the use case described for examination preparation.

The study’s limitations suggest caution in generalizing the findings to other disciplines or educational objectives. Future research could broaden the scope to include diverse medical specialties and different types of educational content to verify the applicability of these results more widely.

### Conclusion

This study reaffirmed the potential of AI, particularly ChatGPT-4, as a powerful tool in medical education, capable of supporting learners in high-stakes examination preparation without the need for specialized enhancements. It highlighted the importance of leveraging the inherent capabilities of advanced AI models and provided a foundation for further research into effective integration strategies in educational settings. This could be accomplished by determining if linking ChatGPT to a dynamic and reliable data source provides benefits, focusing in on highly subspecialized fields with static information sources, and ultimately comparing evaluation and management plans generated by AI to physician counterparts. As AI continues to evolve, its role in education and potentially clinical practice is likely to expand, offering opportunities to enrich learning experiences and access to knowledge.

## Abbreviations

ABEM: American Board of Emergency Medicine
ABFM: American Board of Family Medicine
AI: artificial intelligence
USMLE: United States Medical Licensing Examination
